# Genitourinary Tuberculosis Presenting As Testicular Malignancy and Resulting in Tuberculous Autonephrectomy

**DOI:** 10.7759/cureus.111687

**Published:** 2026-06-28

**Authors:** Viktorija Lodeikina, Liga Kuksa, Iveta Ozere, Reinis Riekstins

**Affiliations:** 1 Tuberculosis and Lung Disease Center, Riga East University Hospital, Riga, LVA; 2 Urology and Urologic Oncology, Riga East University Hospital, Riga, LVA

**Keywords:** extrapulmonary tuberculosis, genitourinary tuberculosis, granulomatous orchitis, nephrectomy, testicular malignancy, tuberculous autonephrectomy, xpert mtb/rif

## Abstract

Genitourinary tuberculosis is a common form of extrapulmonary tuberculosis and may mimic malignancy and chronic bacterial infection, leading to delayed diagnosis and irreversible organ damage. We report a case of genitourinary tuberculosis in a 41-year-old man presenting with chronic epididymal inflammation, a testicular mass, and progressive hydronephrotic destruction of the left kidney. Imaging findings initially raised suspicion for testicular malignancy. Histopathological examination following orchifuniculectomy demonstrated extensive necrotising granulomatous inflammation without evidence of malignancy, while acid-fast bacilli were not identified. Molecular testing of urine samples detected Mycobacterium tuberculosis complex deoxyribonucleic acid (MTBC DNA), and one of several mycobacterial urine cultures subsequently yielded positive growth for Mycobacterium tuberculosis. Imaging demonstrated destructive changes of the left kidney and mild bilateral apical pulmonary lesions compatible with tuberculosis, while sputum and bronchial aspirate microscopy, culture, and Xpert Mycobacterium tuberculosis and Rifampicin (MTB/RIF) testing (Cepheid, Sunnyvale, USA) remained negative. Anti-tuberculosis treatment was initiated. Because of complete functional loss and extensive parenchymal destruction, laparoscopic nephrectomy was required. Histopathological examination of the nephrectomy specimen confirmed necrotising granulomatous inflammation consistent with renal tuberculosis. This case highlights the diagnostic challenges of genitourinary tuberculosis, which is frequently associated with nonspecific symptoms and low mycobacterial load, and emphasises the importance of combining radiological, histopathological, molecular, and microbiological findings.

## Introduction

Genitourinary tuberculosis (GUTB) is one of the most common forms of extrapulmonary tuberculosis, accounting for approximately 20% of extrapulmonary cases worldwide [1]. It typically develops through haematogenous dissemination from a pulmonary focus and may involve the kidneys, ureters, bladder, prostate, epididymis and testes in men, as well as the fallopian tubes, endometrium and ovaries in women [1]. Despite its prevalence, GUTB remains frequently underdiagnosed because of its nonspecific clinical presentation, indolent course, and low mycobacterial burden, which limit the diagnostic yield of conventional microbiological methods and contribute to its tendency to mimic other conditions, including malignancy and chronic bacterial infection [1,2]. Delayed recognition may result in irreversible organ destruction, including complete and permanent loss of renal function, a condition known as tuberculous autonephrectomy, which represents an uncommon but clinically significant end-stage complication of renal tuberculosis. We present a case of GUTB in a 41-year-old immunocompetent man, in whom the disease initially manifested as a testicular mass suspicious for malignancy and ultimately resulted in tuberculous autonephrectomy, highlighting the diagnostic challenges associated with this condition.

## Case presentation

A 41-year-old man presented in February 2025 with acute pancreatitis. His medical history included alcohol-induced pancreatitis, hepatic steatosis, and Purtscher retinopathy. Human immunodeficiency virus (HIV) serology was negative.

Abdominal CT performed during this admission revealed chronic destructive changes of the left kidney, including hydronephrosis, partial renal atrophy, reduced parenchymal enhancement, parenchymal calcifications, multiple calculi, and a distal ureteric stone (Figure 1A). The patient improved with conservative management of pancreatitis and was discharged. Notably, similar renal changes had already been identified on imaging in 2022 (Figure 1B), during an emergency admission for lower abdominal pain and increased urinary frequency. Abdominal CT revealed a calculus in the left ureter as well as a thin, atrophic left renal parenchyma with a dilated collecting system and thickened pelvic wall; the right kidney demonstrated compensatory parenchymal hypertrophy with a non-dilated collecting system and no calculi. Symptoms improved with conservative management at that time as well. Outpatient urological follow-up was recommended but not attended because of poor adherence to follow-up care.

**Figure 1:**
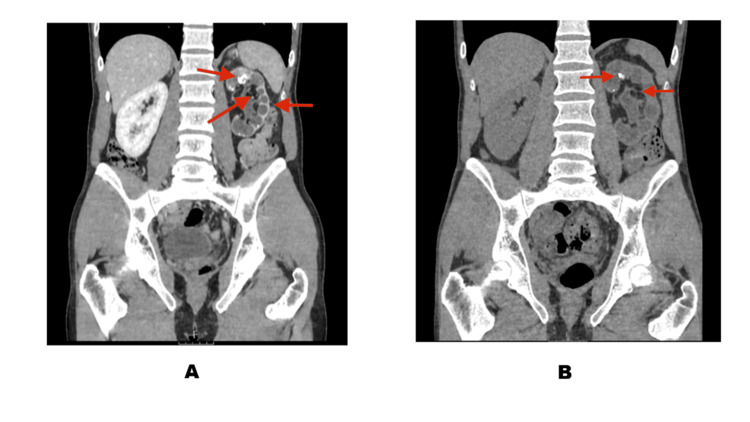
Serial abdominal CT imaging demonstrating progressive destructive changes of the left kidney. (A) Contrast-enhanced CT in the late venous phase obtained in 2025, demonstrating progressive destruction of the left kidney with marked hydronephrotic transformation, parenchymal thinning, and multiple calyceal calculi, most pronounced in the upper pole (arrows); no calculi or evidence of urinary obstruction are seen on the right. (B) Non-contrast CT obtained in 2022 demonstrating a thin, atrophic left renal parenchyma with a dilated collecting system and thickened pelvic wall (arrows); the right kidney demonstrates compensatory parenchymal hypertrophy with a non-dilated collecting system and no calculi.

Shortly after discharge in February 2025, the patient developed pain and induration of the left testis and was seen by a urologist as an outpatient. Urinary symptoms, dysuria, and fever were denied, although a low-grade temperature of up to 37°C was noted. On examination, the left epididymis was firm and tender to palpation, and the clinical presentation was considered consistent with epididymitis. Scrotal ultrasonography, urinalysis, and urine culture were requested, and empirical ciprofloxacin was prescribed for 14 days. Given the chronic renal abnormalities, the urologist also requested dynamic renal scintigraphy to assess renal function.

At follow-up in April 2025, testicular pain had resolved following antibacterial therapy; however, scrotal swelling and induration of the testis persisted. Urine culture was negative, while urinalysis demonstrated leukocyturia and microscopic haematuria. Scrotal ultrasonography demonstrated a heterogeneous lesion involving the left testis and epididymis suspicious for malignancy. Dynamic renal scintigraphy performed in March 2025 demonstrated severely reduced left renal function, with relative renal function of only 4.8%, whereas the right kidney showed preserved compensatory function. 

Following the suspicious ultrasonographic findings, CT imaging of the chest, abdomen, and pelvis was performed in April 2025 as part of standard oncological staging to exclude distant metastatic disease. This revealed mild bilateral apical pulmonary nodules, several with calcified components (Figure 2), enlarged para-aortic lymph nodes, marked hydronephrotic transformation of the left kidney with parenchymal calcifications, and an enlarged left testis and epididymis with heterogeneous and irregular contrast enhancement (Figure 3), corroborating the ultrasonographic findings. 

**Figure 2:**
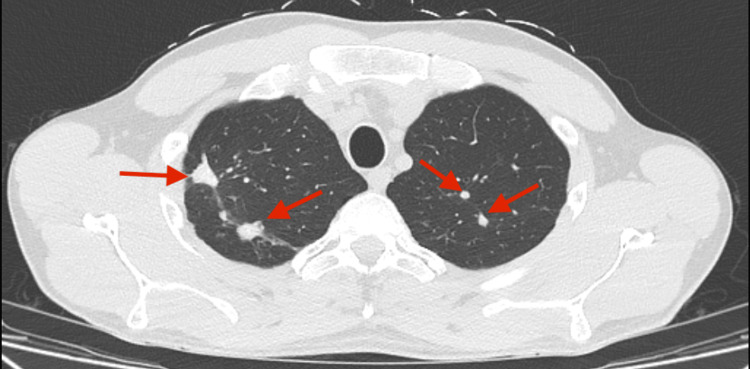
CT imaging of the chest demonstrating mild bilateral apical nodular lesions with calcified foci (arrows).

**Figure 3:**
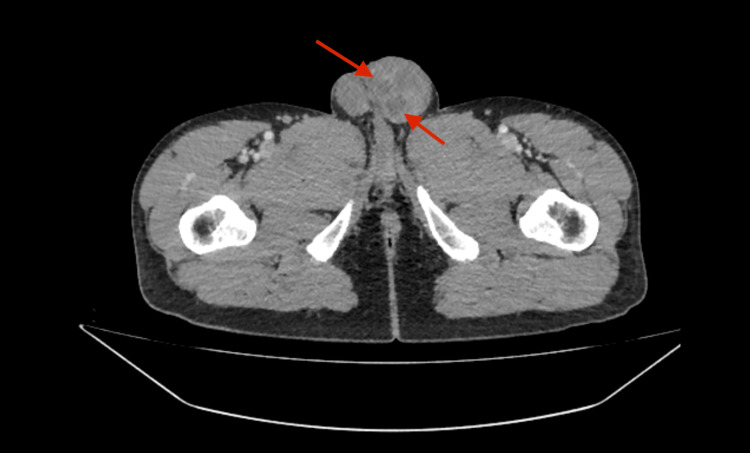
Contrast-enhanced abdominal CT demonstrating an enlarged left testis and epididymis with heterogeneous and irregular contrast enhancement (arrows), raising suspicion for malignancy.

The findings were initially considered consistent with testicular malignancy. The patient underwent left orchifuniculectomy on 11 April 2025.

Histopathological examination revealed extensive necrotising granulomatous inflammation involving nearly the entire testicular and epididymal parenchyma (Figure 4). No malignant cells were identified. Ziehl-Neelsen and Grocott staining did not reveal acid-fast bacilli or fungal structures. The pathological report recommended exclusion of tuberculosis and other causes of granulomatous orchitis.

**Figure 4:**
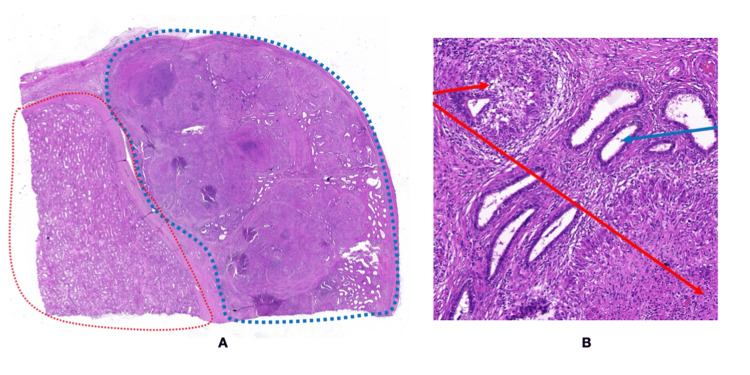
Histopathological examination of the orchifuniculectomy specimen (haematoxylin and eosin stain). (A) Extensive necrotising granulomatous inflammation involving the epididymis (blue outline) and the testicular parenchyma (red outline). (B) A well-formed epithelioid granuloma (red arrow) adjacent to epididymal structures (blue arrow).

The patient was subsequently referred to a specialised tuberculosis centre, where further diagnostic evaluation was undertaken in July 2025. Repeated sputum and bronchial aspirate examinations, including smear microscopy, mycobacterial culture, and Xpert Mycobacterium tuberculosis and Rifampicin (MTB/RIF) testing (Cepheid, Sunnyvale, USA), remained negative for acid-fast bacilli and MTBC DNA.

Xpert MTB/RIF testing of two urine specimens demonstrated trace detection of Mycobacterium tuberculosis complex deoxyribonucleic acid (MTBC DNA). Rifampicin resistance could not be determined because of the low bacillary burden. Of several mycobacterial cultures of urine specimens performed on solid (Löwenstein-Jensen) medium, one culture subsequently yielded positive growth (two colonies) of Mycobacterium tuberculosis, while the remaining specimens showed no growth. Xpert MTB/RIF extensively drug-resistant (XDR) testing performed on this isolate did not detect resistance to isoniazid, ethionamide, fluoroquinolones, amikacin, kanamycin, or capreomycin. The isolate was also subcultured on BACTEC (Becton Dickinson, Franklin Lakes, USA) liquid Mycobacteria growth indicator tube (MGIT) media for drug susceptibility testing, which demonstrated susceptibility to isoniazid, rifampicin, ethambutol, and levofloxacin. Follow-up urine testing no longer detected MTBC DNA. Interferon-gamma release assay testing was positive for sensitisation to Mycobacterium tuberculosis. Results of tuberculosis-specific investigations are presented in Table 1. The urinalysis showed persistent microscopic hematuria and albuminuria.

**Table 1:** Diagnostic investigations for tuberculosis AFB: acid-fast bacilli; MTB/RIF: Mycobacterium tuberculosis and Rifampicin; MGIT: mycobacteria growth indicator tube; MTBC: Mycobacterium tuberculosis complex; XDR: extensively drug-resistant; IGRA: interferon-gamma release assay.

Investigation	Sample	Result	Reference
Sputum smear microscopy (AFB)	Sputum	Negative (x6)	Negative
Mycobacterial culture (BACTEC MGIT)	Sputum	No growth (x3)	No growth
Mycobacterial culture (Löwenstein–Jensen)	Sputum	No growth (x6)	No growth
Xpert MTB/RIF	Sputum	Negative (x4)	Negative
Bronchial aspirate microscopy (AFB)	Bronchial aspirate	Negative (x1)	Negative
Mycobacterial culture (BACTEC MGIT)	Bronchial aspirate	No growth (x1)	No growth
Mycobacterial culture (Löwenstein–Jensen)	Bronchial aspirate	No growth (x1)	No growth
Xpert MTB/RIF	Bronchial aspirate	Negative (x1)	Negative
Urine smear microscopy (AFB)	Urine	Negative (x8)	Negative
Xpert MTB/RIF	Urine	Trace positive (x2); rifampicin resistance indeterminate	Negative
Xpert MTB/RIF XDR	Urine culture isolate	No resistance detected to isoniazid, ethionamide, fluoroquinolones, amikacin, kanamycin or capreomycin	Negative
Mycobacterial culture (Löwenstein–Jensen)	Urine	1 of 8 positive (2 colonies); remaining 7 no growth	No growth
Drug susceptibility testing (BACTEC MGIT)	Urine culture isolate	Susceptible to isoniazid, rifampicin, ethambutol and levofloxacin	Susceptible
IGRA	Blood	Positive	Negative
Ziehl–Neelsen stain	Orchifuniculectomy specimen	Negative	Negative
Ziehl–Neelsen stain	Nephrectomy specimen	Negative	Negative

Repeat CT imaging of the chest in July 2025 demonstrated stable findings, now considered consistent with pulmonary tuberculosis. Magnetic resonance imaging of the pelvis, performed in July 2025, showed fibrotic changes around the left ureteric orifice and distal ureter without evidence of prostatic abscess or malignancy (Figure 5).

**Figure 5:**
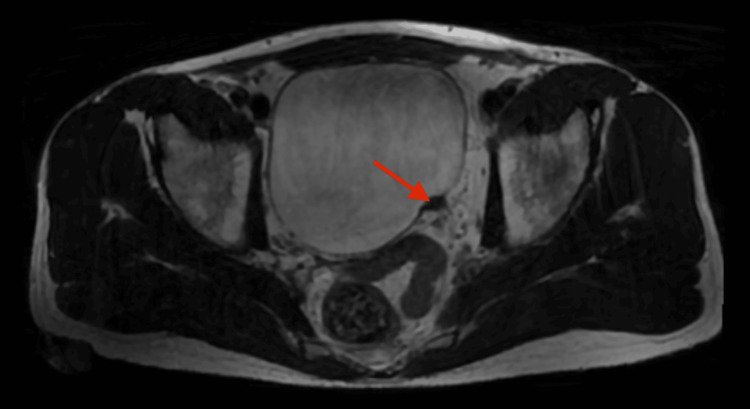
Axial T2-weighted pelvic MRI demonstrating fibrotic changes around the left ureteric orifice (arrow). Image quality is limited by motion artefacts.

The patient was diagnosed with tuberculosis involving the lungs and genitourinary tract. Anti-tuberculosis treatment with isoniazid, rifampicin, pyrazinamide, and ethambutol (HRZE) was initiated on 16 July 2025. Treatment was well tolerated without major adverse effects. Liver function tests, including alanine aminotransferase, aspartate aminotransferase, and bilirubin, were monitored at intervals consistent with local clinical guidelines and remained within normal limits throughout treatment. Serial laboratory parameters, including liver function tests, are presented in Table 2. 

**Table 2:** Laboratory findings at key clinical timepoints Elevated liver enzymes and bilirubin in 2022 are attributed to alcohol-related hepatic steatosis. Elevated CRP and liver enzymes in February 2025 reflect acute pancreatitis rather than tuberculosis activity. GFR: glomerular filtration rate; ALT: alanine aminotransferase; AST: aspartate aminotransferase; WBC: white blood cell count; CRP: C-reactive protein.

Test	2022	Feb 2025	Jul 2025	Apr 2026 (Pre-op)	Reference range	Units
Creatinine	90	85	97	84	62-106	μmol/L
GFR	87.09	-	78.63	92.49	≥90	ml/min/1.73m²
ALT	98.5	57	16.1	59.40	10-50	U/L
AST	-	20	-	-	1-50	U/L
Bilirubin	36.9	15	11.8	6.78	<21.0	μmol/L
Haemoglobin	15.00	13.5	13.9	13.7	14.00-16.00	g/dL
WBC	8.75	10.9	9.64	6.08	4.00-9.00	x 10^9^/L
CRP	3.76	104.22	0.68	1.24	0.0-5.0	mg/L

Given the progressive radiological destruction demonstrated since 2022 and the severely impaired renal function documented on scintigraphy, the left kidney was considered non-functioning and a potential persistent source of infection. Initiation and stabilisation of anti-tuberculosis therapy were prioritised before proceeding with surgical management. Laparoscopic left nephrectomy was performed on 30 April 2026 (Figure 6), while anti-tuberculosis therapy was ongoing, and the postoperative course was uncomplicated.

**Figure 6:**
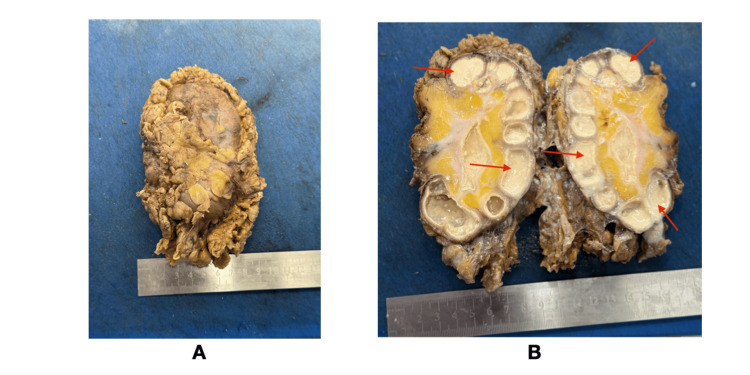
Gross pathological specimen of the left nephrectomy. (A) External view of the kidney demonstrating parenchymal atrophy and cortical thinning. (B) Cut surface demonstrating extensive parenchymal destruction with areas of caseous necrotic material filling the dilated pelvicalyceal system (arrows), consistent with end-stage renal tuberculosis.

Histopathological examination of the nephrectomy specimen demonstrated widespread necrotising granulomatous inflammation replacing nearly the entire renal parenchyma. Multiple well-formed granulomas composed of epithelioid macrophages, multinucleated giant cells, and peripheral lymphocytic infiltrates were identified. Extensive interstitial nephritis and glomerulosclerosis were also present (Figure 7). Ziehl-Neelsen staining remained negative for acid-fast bacilli, and no evidence of malignancy was identified. In the clinical context, the findings were considered consistent with renal tuberculosis.

**Figure 7:**
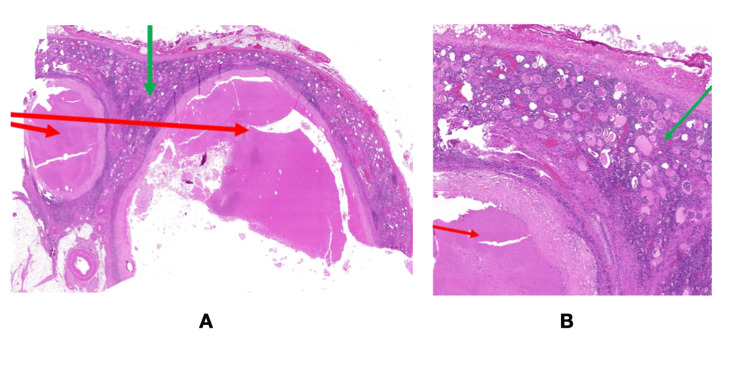
Histopathological examination of the nephrectomy specimen (haematoxylin and eosin stain). (A) Residual renal parenchyma (green arrow) and a well-formed epithelioid granuloma (red arrow). (B) A granuloma with central caseous necrosis (red arrow) surrounded by residual renal parenchyma (green arrow).

Following surgery, the patient has remained clinically stable, with preserved contralateral renal function, and continues to receive anti-tuberculosis therapy, with a total treatment duration of 12 months determined by multidisciplinary tuberculosis consilium decision.

The timeline of key diagnostic and therapeutic events is summarised in Table 3.

**Table 3:** Timeline of clinical events. AFB: acid fast bacilli; IGRA: interferon-gamma release assay; MTB/RIF: Mycobacterium tuberculosis and Rifampicin; MTBC: Mycobacterium tuberculosis complex; HRZE: isoniazid, rifampicin, pyrazinamide and ethambutol; MTBC DNA: Mycobacterium tuberculosis complex deoxyribonucleic acid.

Date	Clinical event	Diagnostic/ therapeutic intervention	Outcome
2022	Emergency admission with lower abdominal pain and increased urinary frequency	Abdominal CT	Left ureteric calculus; chronic destructive renal changes; symptoms improved with conservative management; outpatient urological follow-up recommended but not attended
Feb 2025	Admission with acute pancreatitis	Abdominal CT	Chronic destructive changes of the left kidney confirmed; improved with conservative management; discharged
Feb 2025	Pain and induration of the left testis shortly after discharge	Clinical assessment; additional examinations requested	Diagnosis of epididymitis; ciprofloxacin prescribed.
March 2025	Functional renal assessment.	Dynamic renal scintigraphy.	Left renal function 4.8% (non-functioning kidney).
April 2025	Follow-up: Persistent testicular induration despite antibiotics.	Scrotal ultrasonography.	Suspicion of testicular malignancy.
April 2025	Systematic evaluation for malignancy.	CT Chest/ Abdomen/ Pelvis.	Apical pulmonary nodules, paraaortic lymphadenopathy, and renal destruction identified.
11 April 2025	Surgical intervention.	Left orchifuniculectomy.	Histopathology: Necrotising granulomatous inflammation; AFB negative.
July 2025	Referral to a specialised tuberculosis centre.	Sputum/bronchial aspirate microscopy and culture; urine Xpert MTB/RIF and culture; IGRA testing	Urine Xpert MTB/RIF and culture positive for MTBC; IGRA positive; sputum/bronchial aspirate negative.
16 July 2025	Diagnosis of tuberculosis.	Initiation of anti-TB therapy (HRZE).	Treatment well tolerated; clinical stabilisation.
30 April 2026	Management of end-stage renal destruction.	Laparoscopic left nephrectomy.	Histopathology confirmed renal TB; uncomplicated recovery.
Postoperative	Follow - up	Continuation of therapy; repeat urine Xpert MTB/RIF	Patient stable; urine MTBC DNA no longer detected.

## Discussion

Genitourinary tuberculosis (GUTB) typically develops secondary to haematogenous dissemination from a pulmonary focus and may involve the kidneys, ureters, bladder, prostate, epididymis, and testes. It occurs predominantly in men in the fourth and fifth decades of life [2-4]. Although the patient had a medical history notable for alcohol-induced pancreatitis, hepatic steatosis, and Purtscher retinopathy, he was considered immunocompetent, as HIV serology was negative and no other identifiable cause of immunosuppression was established.

Despite being a well-recognised manifestation of extrapulmonary tuberculosis, GUTB remains a diagnostic challenge because of its non-specific clinical presentation and often low bacterial burden. Patients may present with chronic epididymal inflammation, haematuria, flank pain, or obstructive uropathy, while radiological findings may mimic malignancy or chronic bacterial infection [2].

In the present case, the disease initially manifested as persistent epididymo-orchitis with a heterogeneous testicular mass suspicious for neoplasia. Orchifuniculectomy was therefore performed before tuberculosis was considered. Such presentations are well described in the literature, as tuberculous epididymo-orchitis may be radiologically indistinguishable from testicular malignancy [3,4]. The differential diagnosis of necrotising granulomatous inflammation involving the testis and epididymis includes fungal infections, sarcoidosis, idiopathic granulomatous orchitis, and xanthogranulomatous inflammatory processes. However, these conditions were considered less likely given the concomitant pulmonary abnormalities, chronic destructive renal lesions, a positive urine mycobacterial culture, positive IGRA, positive urine Xpert MTB/RIF findings, and the characteristic histopathological features observed in both surgical specimens. 

The diagnostic course was further complicated by empirical ciprofloxacin therapy prescribed for presumed epididymitis, which failed to result in clinical improvement. Fluoroquinolones exhibit activity against Mycobacterium tuberculosis, and previous studies have suggested that empirical fluoroquinolone treatment may delay the diagnosis of tuberculosis and reduce the microbiological yield of diagnostic investigations by partially suppressing mycobacterial growth. Therefore, tuberculosis should remain an important differential diagnosis in patients with persistent epididymo-orchitis or granulomatous inflammation despite prior antibacterial treatment [5].

Another important feature of this case was the absence of microbiological confirmation in respiratory and histopathological specimens despite extensive organ involvement. Both the orchifuniculectomy and nephrectomy specimens revealed characteristic necrotising granulomatous inflammation; however, Ziehl-Neelsen staining failed to identify acid-fast bacilli. 

Mycobacterial cultures of respiratory specimens yielded no growth on either liquid (BACTEC MGIT) or solid (Löwenstein-Jensen) media. Xpert MTB/RIF likewise showed no detectable MTBC DNA. Only one of several urine cultures showed minimal growth (two colonies) on solid media, reflecting the characteristically low bacterial burden of genitourinary tuberculosis. Nevertheless, the combination of positive Xpert MTB/RIF results, a positive urine culture, and a positive IGRA provided robust microbiological support for the diagnosis [6].

The case also illustrates the destructive consequences of delayed recognition of renal tuberculosis. Retrospective review of the patient's imaging history revealed radiological abnormalities suggestive of renal tuberculosis several years before the diagnosis was established, ultimately leading to irreversible renal destruction. In patients with unexplained chronic destructive renal changes, such as parenchymal calcifications, focal hydronephrosis, or a “putty kidney” appearance, tuberculosis should be actively excluded even in the absence of pulmonary symptoms. In this patient, progressive hydronephrosis and severe functional impairment eventually resulted in complete loss of renal function, a process known as tuberculous autonephrectomy, which represents the end stage of renal tuberculosis and is characterised by caseous necrosis and progressive parenchymal destruction [7]. Although anti-tuberculosis therapy remains the cornerstone of treatment, surgical intervention is still required in selected patients with advanced anatomical destruction, persistent symptoms, or loss of renal function [6].

This case also highlights several limitations that contributed to the delayed diagnosis. First, conventional microbiological methods, including smear microscopy and culture, have inherently low sensitivity in genitourinary tuberculosis due to the characteristic low bacillary burden, which may have been further compounded by prior empirical antibacterial therapy. Second, the insidious and nonspecific nature of genitourinary tuberculosis, which may mimic malignancy or chronic bacterial infection over a prolonged period, delayed clinical suspicion until histopathological findings prompted further evaluation. Third, poor patient adherence to recommended outpatient follow-up after the 2022 finding, partly related to excessive alcohol use, prevented timely evaluation of the renal abnormalities at that time, allowing the disease to progress before being incidentally identified again in 2025 in the context of an unrelated acute presentation. Importantly, once histopathological findings raised suspicion for tuberculosis, the subsequent clinical pathway, including pulmonology referral and multidisciplinary evaluation, proceeded promptly and systematically.

Overall, this case underscores the importance of considering tuberculosis in patients with persistent granulomatous genitourinary disease and unexplained destructive renal lesions, particularly when routine investigations fail to establish an alternative diagnosis. Early multidisciplinary assessment integrating radiological, histopathological, molecular, and microbiological findings is essential to facilitate timely diagnosis and prevent irreversible organ damage.

## Conclusions

Tuberculosis is frequently perceived as a disease of the past, yet it continues to present in unexpected and misleading ways. This case demonstrates that persistent granulomatous inflammation, atypical radiological findings, and an unexpected clinical course should prompt clinicians to actively reconsider tuberculosis, even when conventional acid-fast bacilli staining is negative and mycobacterial culture yield is minimal. In this patient, the diagnosis was confirmed through a combination of molecular, microbiological, immunological, and histopathological findings. Earlier recognition of this pattern may help prevent irreversible complications, including tuberculous autonephrectomy.
